# Production of bone mineral material and BMP-2 in osteoblasts cultured on double acid-etched titanium

**DOI:** 10.4317/medoral.22071

**Published:** 2017-08-16

**Authors:** Rocío Velázquez-Cayón, Gabriel Castillo-Dalí, Jose-Ramón Corcuera-Flores, María-Ángeles Serrera-Figallo, Raquel Castillo-Oyagüe, Maribel González-Martín, Jose-Luis Gutierrez-Pérez, Daniel Torres-Lagares

**Affiliations:** 1 DDS, PhD, Master’s Degree in Oral Surgery. School of Dentistry. University of Seville; 2 DDS, PhD, Associate Professor, Master’s in Integrated Dentistry and Patients with Special Diseases. School of Dentistry. University of Seville; 3 DDS, PhD, Associate Professor, PhD in Stomatological Prosthesis. School of Dentistry of the Complutense University of Madrid; 4 DMD, PhD, Affiliated Professor of the Master’s Degree in Oral Surgery. School of Dentistry. University of Seville; 5 DDS, PhD, Professor of the Master’s Degree in Oral Surgery. School of Dentistry. University of Seville

## Abstract

**Background:**

The study of osteoblasts and their osteogenic functions is essential in order to understand them and their applications in implantology. In this sense, this study try to study BMP-2 production and bone matrix deposition, in addition to other biological variables, in osteoblasts cultured on a rough double acid-etched titanium surface (Osseotite®, Biomet 3i, Palm Beach Garden, Florida, USA) in comparison to a smooth titanium surface (machined) and a control Petri dish.

**Material and Methods:**

An *in vitro* prospective study. NHOst human osteoblasts from the femur were cultured on three different surfaces: Control group: 25-mm methacrylate dish (n = 6); Machined group: titanium discs with machined surface (n = 6) and Experimental group: titanium discs with a double acid-etched nitric and hydrofluoric Osseotite® acid surface (n = 6). A quantification of the mitochondrial membrane potential, and studies of apoptosis, mobility and adhesion, bone productivity (BMP-2) and cellular bone synthesis were carried out after culturing the three groups for forty-eight hours.

**Results:**

A statistically significant difference was observed in the production of BMP-2 between the experimental group and the other two groups (22.33% ± 11.06 vs. 13.10% ± 5.51 in the machined group and 3.88% ± 3.43 in the control group). Differences in cellular bone synthesis were also observed between the groups (28.34% ± 14.4% in the experimental group vs. 20.03% ± 6.79 in the machined group and 19.34% ± 15.93% in the control group).

**Conclusions:**

In comparison with machined surfaces, Osseotite® surfaces favor BMP-2 production and bone synthesis as a result of the osteoblasts in contact with it.

** Key words:**BMP-2, Cytoskeleton, cell culture, bone matrix, apoptosis, cell viability.

## Introduction

The concept of osseointegration, the basis of implantology, should be defined on multiple levels: clinical, anatomical, histopathological and ultrastructural. The term osseointegration was first introduced by Bränemark *et al.* in 1969, although it was defined by Albrektsson *et al.* in 1981 (1) as “the direct, structural and functional connection between living bone and the surface of a functionally loaded endosseous implant.” It results in a direct mechanic union that is stable and without any interposition of connective tissue, recognizable with an optical microscope and thus immobile. Subsequently, other authors proposed a definition that references a much more clinical concept, which considers osseointegration as a process in which the clinically asymptomatic rigid fixation of alloplastic material is achieved and maintained in the bone during functional loading (2).

Osseointegration is achieved with implants made of bioinert materials such as titanium. However, osseointegration does not depend exclusively on the material’s biocompatibility, but also on correct implant fitting in the bone, the surgical technique used, implant design, and quality of bone tissue.

Osteoblasts, differentiated cells that synthesize collagen and bone substance, play a primary role in this process. Osteoblasts can become osteocytes, surrounded by the growing bone matrix, or they can remain at the surface of the newly formed bone tissue, where they flatten to form a bony covering.

Bone tissue is primary formed by bone matrix, which contains mineral salts deposited in form of hydroxyapatite crystals or tricalcium phosphate in fibers mainly formed of collagen and bone cells (osteoblasts, osteoclasts, osteocytes and osteogenic cells). Osteoblasts are large cells (20-30 μm) responsible for bone formation and maintaining skeletal architecture. Their main function is the synthesis and deposition of proteins in the osteoid matrix (3,4).

In order to understand osseointegration, the primary mechanism behind successful dental implants, the underlying mechanisms that make it possible must first be understood, among them cellular adhesion, especially that of osteoblasts.

The study of osteoblasts and their osteogenic functions is essential in order to understand them and their applications in implantology *in vitro* experiments are used to study them and other cells, with *in vivo* verification carried out after collecting rigorous data. The more advanced the *in vitro* studies, the more data collected and the potential fewer errors that can be made during *in vivo* experimentation. In this sense, bone deposition by osteoblasts has never before been studied *in vitro* on titanium discs following the protocol adopted in the present study.

Cytoskeletons are often found in eukaryotic cells, complex cells that have to organize their organelles, move their vesicles within the cytoplasm, and even move themselves. These functions are accomplished thanks to the intermolecular cytoskeletal system, which is made up of three different types of filaments: actin microfilaments, intermediate filaments and microtubules (5,6).

Physicochemical properties such as surface energy, surface charge, and surface composition can be manipulated by using different surface treatments to obtain better interaction between implants, cells, and surrounding tissues (7-10).

After implant placement, two phenomena may take place at the surface of the implant. The bone may come into contact with the surface and the necessary mechanisms may occur, resulting in osseointegration or, alternatively, a fibrous encapsulation may form, resulting in failed treatment. That is why it is important to differentiate the topographical features of the implant’s surface, distinguishing between smooth and rough surfaces.

A rough titanium surface treated with double acid etching was chosen to analyze the behavior of osteoblasts, since the latest studies on animals have shown its promising levels of bone implant surface adherence (11).

Human bone morphogenetic proteins (BMPs) are a subtype of transforming growth factor beta (TGF-β). Within the functions of BMP-2, their presence is correlated with the differentiation of chondroblasts and osteoblasts from pluripotent mesenchymal stem cells, evidencing their osteoinductive function (12-14).

The present study arises from the need to better understand the behavior of osteoblasts and their osteogenic function as the main type of cell involved in osseointegration (15,16). In this regard, studying bone deposition *in vitro* may appear to be an interesting step. The objective is to obtain data about their *in vitro* behavior in order to contribute to published scientific evidence in a rigorous manner, and as a stepping-stone from which to develop a better understanding of the behavior of osteoblasts *in vitro* and its clinical applications.

## Material and Methods

An *in vitro* prospective study was conducted by the University of Seville’s Research Technology and Innovation Center. Ethical Committee of the University of Seville has authorized the protocol of this study. To study the overall cell performance of bone-forming cells (osteoblasts) on different implant surfaces, several types of titanium discs with smooth or machined surfaces were used (Biomet 3i, Palm Beach Garden, Florida, USA; n = 6), in addition to some titanium discs treated with a double nitric and hydrofluoric acid-etched surface (dual acid-etched, DAE, Osseotite®, Biomet 3i, Palm Beach Garden, Florida, USA; n = 6). Both types of disc were supplied by Biomet 3i (Palm Beach Garden, Florida, USA). Methacrylate petri dishes of 25 mm were also used as controls in the experiment (n = 6).

- Microstructural characterization

**Figure 1:**
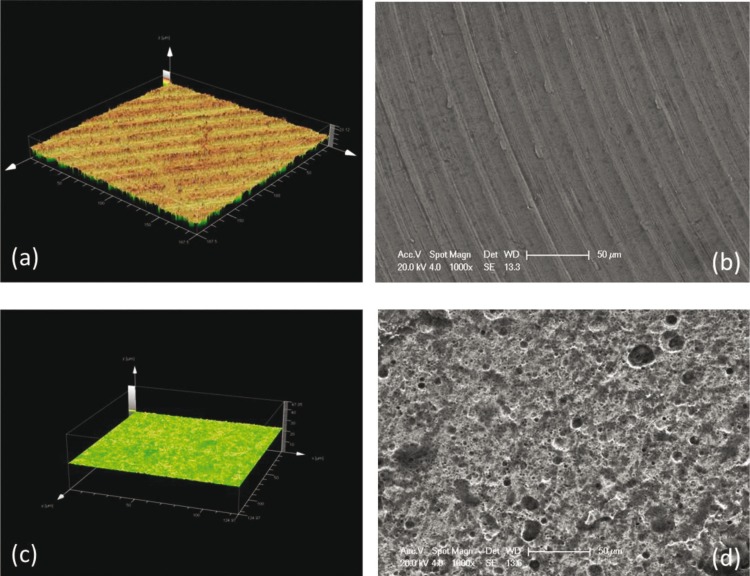
Image of the microstructural characterization of titanium discs captured with a confocal microscope in flex mode [machined disc 3D map (a) and Ossoeotite® disc (c)] and a Philips XL30-II at 1000x scanning electron microscope [machined disc (b) and Ossoeotite® disc (d)].

A Philips XL30-II scanning electron microscope (Amsterdam, The Netherlands) was used under high vacuum to visualize titanium discs with a machined surface (Biomet 3i, Palm Beach Garden, Florida, USA) and titanium discs with a double nitric and hydrofluoric acid-etched surface (dual acid-etched, DAE, Osseotite®, Biomet 3i, Palm Beach Garden, Florida, USA), without the need for sputtering and 3D-mapping with a confocal microscope in reflection mode (Fig. 1).

- Cell culture 

NHOst human bone cells from the femur were cultured in both petri dishes and the discs provided by pharmaceutical laboratory Lonza Biologics (O Porriño, Pontevedra, Spain). A laminar flow cabinet (LHC-4A model) was also used.

A DMEM culture medium exclusively for osteoblasts was used, with (L-glutamine / sodium pyruvate) 500 ml (4.5 g/L) (PAA Cell Culture Company, BioPathStores, Cambridge, United Kingdom), L-glutamine (5 ml), fetal bovine serum (FBS) (50 ml) (MP Biomedicals, Santa Ana, CA, USA) supplemented with antifungal and antibiotic treatments (penicillin, amphotericin B, streptomycin) (5 ml).

A Sigma refrigerated centrifuge, model 3K-30, (SciQuipLtd, Newtown, Wem, Shropshire, United Kingdom) was used for the gravimetric separation of cells and cellular components. A laboratory oven or incubator was used to grow cell cultures (Sanyo Electrical Biomedical Co., Osaka, Japan). All osteoblasts were cultured at a temperature of 98.6º F (37º C), with 95% humidity and 5% CO2 for 48 hours. Cellular growth control was carried out using an Olympus CKX41 optical microscope (Olympus Corporation, Shinjuku, Tokyo, Japan) on both the cell culture flask and the osteoblasts seeded onto culture trays prior to being marked with fluorescent colors.

The following analyses of this sample were performed:

- Cellular energy analysis: a quantification of the mitochondrial membrane potential was carried out using JC-1 staining (5,5’,6,6’-tetrachloro-1,1’,3,3’-tetraethylbenzimidazolylcarbocyanine iodide). The results were used to assess the amount of ener-gy said cell is capable of releasing, which is subsequently stored as ATP and used to maintain cellular activity (17).

JC-1 is a lipophilic monomeric dye that stains the cytosol green. When the membrane potential increases (becoming more active) due to the high density of intermembrane proteins, which generate a physicochemical gradient in mitochondria, thereby facilitating the infiltration of this marker into the mitochondrial matrix, where they aggregate in the form of red dimers (18).

The preparation consisted of 5 mg/ml of JC-1 stock solution (Merck KGaA, Darmstadt, Germany) in dimethyl sulfoxide (DMSO 7.7 nm) (Sigma-Aldrich Co. St. Louis, USA). They were kept away from direct sunlight, stained under low light and incubated under aluminum foil.

After staining, images were obtained using a confocal microscope (Leica TCS-SP2; Leica Microsystem, Wetzlar, Germany) with a general Argón laser potentiometer at 50%, with a 40x objective and a x2.3 zoom. The images were acquired with a z-scan in ten planes and four seconds apart to help localize the best focal plane. Once obtained, the region of interest (ROI) for each region was marked, and the options for automatic count of red/green pixels and percentage (%) of each area were selected.

- Apoptosis analysis: fluorescent staining of cellular nuclei using DAPI (4’,6-diamidino-2-phenylindole).

This method has been used to assess cell viability, osteoblast capacity for self-elimination when no longer needed, in order to provide space for new cells.

4’,6-diamidino-2-phenylindole dihydrochloride (DAPI) is a fluorescent stain used to stain DNA, nuclei, and living cells, and to counterstain immunofluorescent stains in human and botanical material, in addition to its use in flow cytometry (19,20).

DAPI contains three different peak absorption spectrums; however, the most important for cytofluorometric analysis is the 340-nm wavelength. DAPI is not optically active on its own, but nucleic acids induce a positive extrinsic Cotton effect that can be captured.

After staining, images were captured using the Olympus BX61® fluorescent light microscope (Olympus Corporation, Shinjuku, Tokyo, Japan) and the AxioScope® camera (Carl Zeiss®, OPM, Pico Dental, Germany) with a 20x objective and a x2.3 zoom. The images were acquired with a ten-plane z-scan, four seconds apart in order to find the best focal plane. Once the images were collected, the regions of interest (ROI) were marked for each area. Subsequently, the number of cells per slide and the number of viable cells vs. apoptotic cells were counted, estimating the average number of viable or apoptotic cells per slide for each type of implant surface.

This is a proven technique described and used in different studies, having been used ever since its introduction (21). It is frequently used today to study the apoptosis of different cells (22,23), especially in the field of medical oncology (24).

- Cytoskeleton analysis: staining with specific anti-actin human antibodies marked with rhodamine-phalloidin.

This technique has been used to evaluate changes in actin cytoskeleton morphology and its migration to adhere better to the surfaces observed in this study.

It is an important study, as low mobility and adherence of the cytoskeleton leads to implant failure (25).

The present study was conducted to have a better comprehension of how osteoblasts adhere to the studied surfaces, in which it plays a major role during the initial phases of adherence, organization, and arrangement of the actin cytoskeleton (26).

Paraformaldehyde 10% was prepared by diluting 1g of paraformaldehyde in 10ml of H2O heated to 60ºC, after which a drop of l M NaOH was added and the mixture was heated until it had dissolved completely. Afterwards, it was filtered with a 0.2-µm filter and diluted in 4% phosphate-buffered saline (Na2HPO4, PBS) for cell-fixation assay.

The samples were prepared for microscopy using a mounting and preserving method for fluorescence by Vectashield® (Vector Laboratories, Inc., Burlingame, CA, USA) or a 10% solution of glycerol (maximum absorption-emission spectrum of 540/565 nm).

The quantification was undertaken in the following manner: after assembling the discs and capturing the images using the fluorescence microscope (Olympus Corporation, Shinjuku, Tokyo, Japan) and AxioScope® camera (Carl Zeils®, OPM, Pico Dental, Germany), a TRITC mercury detector lamp with a 20x objective was used to form a ROI using the IMAGE-J Plus program for each identified cell. The cytoskeleton lengths (in µm) of each cell of the studied areas were recorded in a data sheet to obtain the mean measurements of each study group.

- Bone productivity analysis: BMP-2 protein-specific antibody marker.

This analysis measures BMP-2 production by cells. BMP-2 is a precursor of numerous amino acids that later differentiate into new osteoblasts, which helps to increase the number of cells in contact with titanium (27,28).

The first step of BMP-2 detection was cell fixation, for which 4% formaldehyde (PFA) in PBS was used for one hour, after which it was washed three times with temperature-regulated PBS with neither Ca2+/Mg2+. Each washing lasted one minute. Afterwards, it was immersed in Triton x-100 for thirty minutes in order to facilitate membrane permeation, it was then washed three times with PBS, for one minute each wash.

The sample was incubated with anti-human antibody BMP-2 (Merck KGaA, Darmstadt, Germany) for one hour; it was then washed three times with PBS, with each wash lasting one minute. Finally, the anti-human Fc BMP-2 antibodies were marked using Cys-5 (Merck KGaA, Darmstadt, Germany) for one hour. After cleaning the samples with albumin (it was washed two times using 1% BSA in PBS for 10 minutes), the samples were prepared for examination under the microscope. The Vectashield® mounting and preserving methods for fluorescence (Vector Laboratories, Inc., Burlingame, CA, USA) or a 10% solution of gly-cerol were used.

After mounting the discs, the confocal microscope (Carl Zeils®, OPM, Pico Dental, Germany) was used to capture the images using the 635 helium-neon laser at 50% and a 10x objective. Once all the digital images of each surface were collected, the ROI pixels of each studied surface were automatically quantified, and the total percentage (%) of marked proteins against the black pixels was calculated.

- Analysis of cellular bone synthesis using the Osteoimage TM® method

Mineralization Assay

The main function of this experiment was to quantify the new production of hydroxyapatite (the main component of bone mineralization) by osteoblasts, which allows bone tissue surrounding titanium surfaces to differentiate and mature (29).

Bone mineralization was evaluated using OsteoImage TM staining (OI) (LonzaPoietics TM, Switzerland). OsteoImage TM staining is based on the specific union of an OsteoImage TM fluorescent agent with the hydroxyapatite crystals deposited by osteoblasts. The culture medium used in this study had to be supplemented with calcium, and the culture period was increased up to four weeks in order to allow for the synthesis of such crystals.

The specimens were incubated in the OsteoImage TM solution, which was prepared according to the manufacturer’s instructions, for 30 minutes at room temperature and kept away from light, before being rinsed twice for five minutes each rinse.

The images were acquired using a Zeiss confocal microscope (Carl Zeiss®, OPM, Pico Dental, Germany). They were then used to calculate the average percentage (%) of synthesized bone mineral in each type of titanium disc and the control specimens (Image J Pro 2012, National Institute of Health).

- Statistical analysis

First of all, the individual values of descriptive statistics were obtained (mean, standard deviation). Subsequently, a statistical analysis was carried out between groups to observe their statistical significance. The Kolmogorov-Smirnov test was used to confirm normality, and a comparison of the mean values between groups was carried out using the ANOVA test and applying the Bonferroni post-hoc test.

## Results

**Figure 2:**
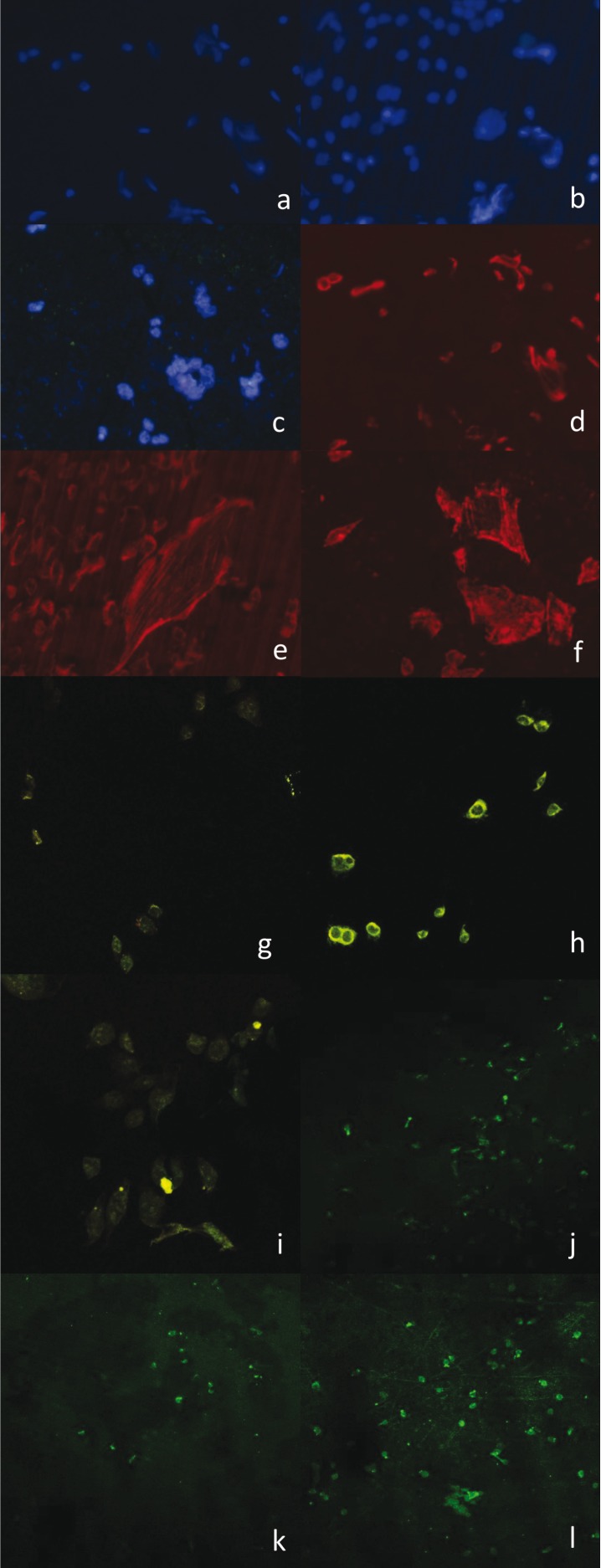
Fluorescence images: (a) Visualization of the nuclei of viable osteoblasts from the Control Group. Obtained with the Olympus BX61® fluorescence light microscope (Olympus Corporation, Shinjuku, Tokyo, Japan) and AxioScope® camera (Carl Zeiss®, OPM, Pico Dental, Germany) with a 20x objective. (b) Visualization of viable osteoblast nuclei of cultures on a machined surface. Images obtained with the Olympus BX61® fluorescence light microscope (Olympus Corporation, Shinjuku, Tokyo, Japan) and AxioScope® camera (Carl Zeiss®, OPM, Pico Dental, Germany) with a 20x objective. (c) Visualization of viable osteoblast nuclei of cultures on an Osseotite® surface. Images obtained with the Olympus BX61® fluorescence light microscope (Olympus Corporation, Shinjuku, Tokyo, Japan) and AxioScope® camera (Carl Zeiss®, OPM, Pico Dental, Germany) with a 20x objective. (d) Visualization of osteoblast cytoskeleton using rhodamine stain of cultured osteoblasts belonging to the Control Group. Images obtained with the Olympus BX61® fluorescence light microscope (Olympus Corporation, Shinjuku, Tokyo, Japan) and AxioScope® camera (Carl Zeiss®, OPM, Pico Dental, Germany) with a 20x objective. (e) Visualization of osteoblast cytoskeleton using rhodamine stain of osteoblasts cultured on a machined surface. Images obtained with the Olympus BX61® fluorescence light microscope (Olympus Corporation, Shinjuku, Tokyo, Japan) and AxioScope® camera (Carl Zeiss®, OPM, Pico Dental, Germany) with a 20x objective. (f) Visualization of osteoblast cytoskeleton using rhodamine stain of osteoblasts cultured on an Osseotite® surface. Images obtained with the Olympus BX61® fluorescence light microscope (Olympus Corporation, Shinjuku, Tokyo, Japan) and AxioScope® camera (Carl Zeiss®, OPM, Pico Dental, Germany) with a 20x objective. (g) Visualization of the Control Group cultured NHOst mitochondria with JC-1 visualized using the LEICA TCS-SP2 laser scanning confocal microscope (Leica Microsystems, Wetzlar, Germany) at 40x. (h) Visualization of NHOst mitochondria cultured on titanium discs with machined surface and stained with JC-1, visualized using the LEICA TCS-SP2 laser scanning confocal microscope (Leica Microsystems, Wetzlar, Germany) at 40x. (i) Visualization of NHOst mitochondria cultured on titanium discs treated with a double acid-etched nitric and hydrofluoric acid surface stained with JC-1 and visualized using the LEICA TCS-SP2 laser scanning confocal microscope (Leica Microsystems, Wetzlar, Germany) at 40x. (j) Visualization of BMP-2 digital images produced by cultured osteoblasts of the Control Group. Images obtained using a confocal microscope (Carl Zeiss®, OPM, Pico Dental, Germany) using a 635 helium-neon laser at 50%, with a 10x objective. (k) Visualization of BMP-2 digital images produced by osteoblasts cultured on a machined surface. Images obtained using a confocal microscope (Carl Zeiss®, OPM, Pico Dental, Germany) using a 635 helium-neon laser at 50%, with a 10x objective. (l) Visualization of BMP-2 digital images produced by osteoblasts cultured on an Osseotite® surface. Images obtained using a confocal microscope (Carl Zeiss®, OPM, Pico Dental, Germany) using a 635 helium-neon laser at 50%, with a 10x objective.

**Figure 3:**
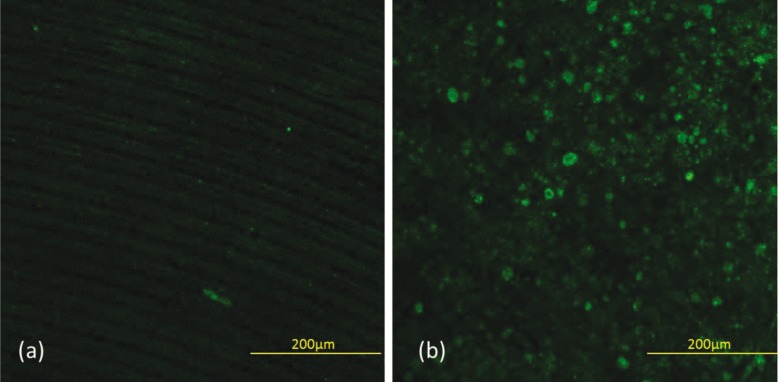
Images of mineral bone material obtained using the Zeiss confocal microscope (Carl Zeiss®, OPM, Pico Dental, Germany). 20x objective. (a) Machined surface. (b) Osseotite ® surface.

Five experiments were carried out (as described above) to assess the behavior of osteoblasts: cellular energy analysis: quantification of mitochondrial membrane potential, apoptosis analysis, mobility and adhesion analysis, and bone productivity and bone synthesis analysis using the OsteoImage TM® method; the corresponding descriptive data are shown in  Table 1. Similarly, the images obtained using various marking techniques are shown in Figures 2 and 3.

**Table 1:**
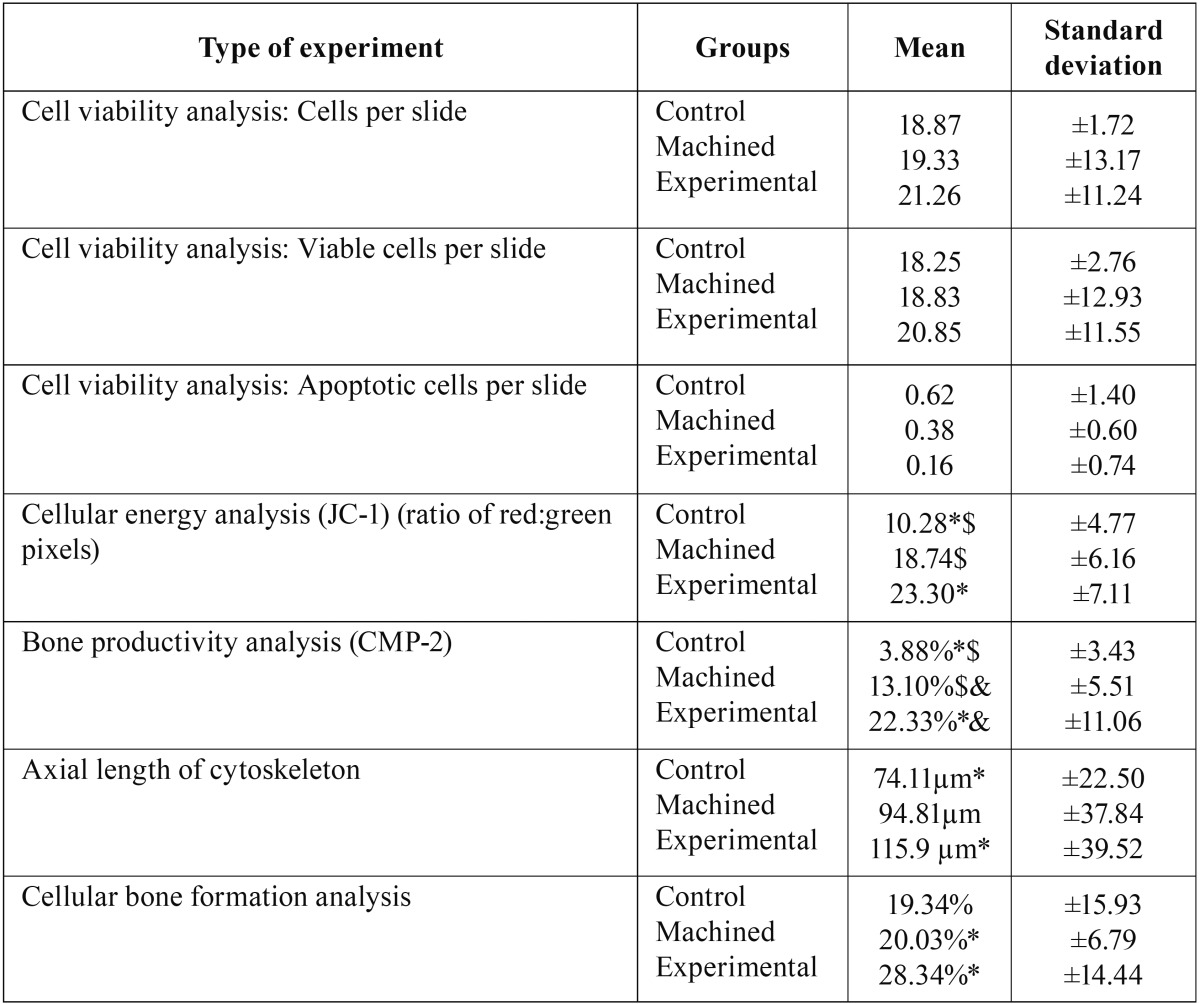
Descriptive data of the *in vitro* experiments carried out. In the same line, the pairs of symbols (*, $, &) indicate statistically significant differences (*p* < 0.05).

Afterwards, the results were compared across the groups for each of the studied variables, finding statistically significant differences in the analysis of bone productivity (specific anti-human BMP-2 protein antibody marker and detection of hydroxyapatite crystals), bone mobility and adhesion analysis (staining with specific anti-human antibodies stained with rhodamine-phalloidin), and cellular energy analysis (quantification of mitochondrial membrane potential via JC-1 staining). This difference was not observed in the apoptosis study with fluorescent staining of cell nuclei using DAPI, in which apoptotic and viable cells were observed and the number of cells per slide was counted and recorded.

The results of this study showed that double acid-etched surfaces (Osseotite®, Biomet 3i, Palm Beach Garden, Florida, USA) favor higher osteoblast production of BMP-2, which is directly correlated with greater bone productivity on these surfaces. The incidence of expression of BMP-2 was found to be 22.3% in the experimental group, 13.1% in the machined group, and only 3.8% in the control group, with a value of *p* < 0.001.

Similarly, improved levels of mobility, adhesion, and cellular energy were observed in the experimental group. The axial length of the osteoblast cytoskeletons was measured, with an average of 115.9 µm in the experimental group, 94.81 µm in the machined group, and 74.11 µm in the control group. There was statistically significant improvement in osteoblast behavior on Osseotite® surfaces in comparison with the control group (*p* = 0.042), but not when compared with the machined group (*p* = 0.526).

The cellular energy analysis also found improved functioning of the osteoblasts in the experimental group. The ratio of red to green pixels is greater in this group, with a value of 23.3, compared to 18.74 in the machined group and 10.28 in the control group. Statistically significant differences were observed between the control and machined groups (*p* = 0.0034) and the control and experimental groups (*p* < 0.001), but not between the machined and experimental groups (*p* = 0.449). Statistically significant differences were found in cellular bone synthesis between the machined and experimental groups (*p* < 0.001).

## Discussion

The present study found significant differences in the production of BMP-2; the experimental group was found to have greater levels of BMP-2 production in comparison to the other groups. Correlating the data reveals the same initial number of osteoblasts will perform better in cultures on Osseotite® surfaces, producing more BMP-2 and therefore exhibiting higher levels of bone production.

OsteoImage TM® staining confirmed that, under the experimental conditions detailed earlier, synthesis of mineralized bone matrix was higher in the experimental group than the machined group. To the authors’ knowledge, this type of experiment on titanium discs and implant surfaces has not appeared in the literature before.

 Moreover, it would appear that only two studies found in the literature specifically analyze BMP-2 production by osteoblasts *in vitro*, although the use of exogenous BMP-2 in *in vitro* studies on titanium discs is frequently mentioned in the literature (30,31).

In the study by Tiainen *et al.* (31), which was carried out in culture conditions similar to those used in the present study and which analyzed the cultures over a period of fourteen days, TiO2 discs that had been treated with hydrofluoric acid were compared with untreated TiO2 discs, finding that the treated discs saw greater expression of BMP-2 than the untreated group.

As in the present study, in which a BMP-2 production level of 22% was observed in the experimental group, as opposed to only 13% BMP-2 production in the untreated disc group, the study by Tiainen *et al.* (31) found that the treated discs saw almost twice the amount of BMP-2 when compared with the control group (1 for the control group, 1.87 in the group immersed in HF for 60 seconds). These are similar results, which reinforces that treated titanium results in higher levels of BMP-2 compared to untreated titanium.

One of the limits of Tiainen *et al.*’s study (31) is that BMP-2 production is expressed as a ratio, with 1 being the value assigned to the untreated titanium group. The ratio is used to calculate the amount of BMP-2 produced by a test group.

In the present study, the amount of BMP-2 produced is expressed as a percentage of expression within each ROI, in an effort to ensure the results can be compared with results of future studies.

Oliveira *et al.*’s study (30) used fetal osteoblast precursor cells cultivated on machined titanium (control group) and on ac-id-etched and anodized titanium surfaces (cultivated for 3 and 7 days at 37ºC and 5% humidity), finding that BMP-2 production was higher after 7 days than it had been at the 3-day mark. However, no statistically significant differences were found between the three kinds of titanium studied, unlike in the present study, which found that Osseotite® surfaces are superior to the other two surfaces studied insofar as the stimulation of osteoblasts to produce BMP-2.

There are already several *in vitro* studies that compare the effectiveness of exogenous BMP-2 at stimulating the production of osteoblasts on a variety of surfaces. A study by Sahrmann *et al.* (32) assessed the viability and proliferation of osteoblast precursor cells cultured on sandblasted, acid-etched, large-grit (SLA) titanium discs; the discs were divided into a case group, which was exposed to a BMP-2 gel, and a control group that was given no such exposure. Osteoblast proliferation was found to be significantly higher in the group treated with BMP-2.

Similar studies have been performed using BMP-2 to observe the behavior of osteoblast precursor cells on discs made of various other metals. For example, a study by Han *et al.* (33) cultivated osteoblast precursor cells on titanium and zirconium discs, which were then stimulated with a gel made up of demineralized bone matrix with BMP-2. After seven days, osteoblast proli-feration was greater in the zirconium group than in the titanium group (*p* < 0.05), which is very favorable result for studies focusing on zirconium implants (34-36).

On the other hand, after quantifying the axial length of the cytoskeleton, the present study found statistically significant differences between the control and experimental groups (*p* = 0.042), but not between the experimental and machined groups (*p* > 0.05); the reason being that the actin cytoskeleton is of a longer length, which indicates it has greater mobility of actin fibers organized in and around the surface, without the presence of stress fibers; this generally results in an increase in contact surface between osteoblast cells and the titanium surface, thereby encouraging their osseointegration (37,38). However, actin cytoskeletons observed in osteoblasts cultured on Osseotite® surfaces show better fiber organization (better cohesion between the same actin fibers when observed under an optical microscope; however, a few stress fibers were observed in the machined group) than machined titanium cultures. These results are similar to the findings of Salido *et al.*’s study (26).

An *in vitro* study performed by Galli *et al.* 2012 (39) cultivated osteoblast precursor cells (MC3TE-E1) on SLA-surface titanium discs and polished titanium discs, comparing changes in cell cytoskeletons of both mediums after 72 hours. They found that osteoblasts cultured on SLA titanium discs had a polygonal, star-shaped morphology with greater elongation of the cytoskeleton and without the appearance of the stress fibers found in the polished titanium group, which also showed a shorter cytoskeleton.

The present study did not find significant differences in the morphology and cytoskeletal elongation of the two titanium surfaces, although the present study made the comparison after only 48 hours rather than the 72 hour time period utilized in Galli *et al.*’s study (39).

As for the cellular energy study, Salido *et al.*’s research (26) used JC-1 mitochondrial staining to determine a red/green ratio of 79.55 ± 28.79 in the experimental group (Osseotite ®) and 84.59 ± 46.74 in the machined group, with a p-value of 0.000 for comparison of Osseotite and machined surfaces. Meanwhile, the present study’s findings were not statistically different (*p* = 0.449).

## Conclusions

Osteoblasts cultured on Osseotite surfaces produce greater levels of BMP-2 and mineral bone material, thereby resulting in greater bone production. This data constitute a fundamental first step in the direction of better understanding the behavior of BMP-2 *in vivo*, with a view to ensuring that dental implant procedures are more stable and performed with greater rigor.

## References

[1] Albrektsson T, Brånemark PI, Hansson HA, Lindström J (1981). Osseointegrated titanium implants. Requirements for ensuring a long-lasting, direct bone-to-implant anchorage in man. Acta Orthop Scand.

[2] Albrektsson T, Sennerby L (1991). State of the art in oral implants. J Clin Periodontol.

[3] Pérez-Sayáns M, Somoza-Martín JM, Barros-Angueira F, Rey JM, García-García A (2010). RANK/RANKL/OPG role in distraction osteogenesis. Oral Surg Oral Med Oral Pathol Oral Radiol Endod.

[4] Özan F, Çörekçi B, Toptaş O, Halicioğlu K, Irgin C, Yilmaz F (2015). Effect of Royal Jelly on new bone formation in rapid maxillary expansion in rats. Med Oral Patol Oral Cir Bucal.

[5] Schmit A, Hall MN (1998). Signalin to the actin cytoskeleton. Annu Rev Cell Dev Biol.

[6] Strelkov S, Herrmann H, Geisler N, Wedig T, Zimbelmann R (2002). Conserved segments 1A and 2B of the intermediate filament dimmer: their atomic structures and role in filament assembly. The EMBO Journal.

[7] Le Guehennec L, Soueidan A, Layrolle P, Amouriq Y (2007). Surface treatments of titanium dental implants for rapid osseointegration. Dent Mater.

[8] Pattanaik B, Pawar S, Pattanaik S (2012). Biocompatible implant surface treatments. Indian J Dent Res.

[9] Louropoulou A, Slot DE, Van der Weijden FA (2012). Titanium surface alterations following the use of different mechanical instruments: a systematic review. Clin Oral Implants Res.

[10] Lozano-Carrascal N, Salomó-Coll O, Gilabert-Cerdà M, Farré-Pagés N, Gargallo-Albiol J, Hernández-Alfaro F (2016). Effect of implant macro-design on primary stability: A prospective clinical study. Med Oral Patol Oral Cir Bucal.

[11] Coelho PG, Zavanelli RA, Salles MB, Yeniyol S, Tovar N, Jimbo R (2016). Enhanced Bone Bonding to Nanotextured Implant Surfaces at a Short Healing Period: A Biomechanical Tensile Testing in the Rat Femur. Implant Dent.

[12] Centrella M, Horowitz MC, Wozney JM, McCarthy TL (1994). Transforming growth factor-beta gene family members and bone. Endocr Rev.

[13] Díaz-Sánchez RM, Yáñez-Vico RM, Fernández-Olavarría A, Mosquera-Pérez R, Iglesias-Linares A, Torres-Lagares D (2015). Current Approaches of Bone Morphogenetic Proteins in Dentistry. J Oral Implantol.

[14] Yu Q, He S, Zeng N, Ma J, Zhang B, Shi B (2015). BMP7 Gene involved in nonsyndromic orofacial clefts in Western han Chinese. Med Oral Patol Oral Cir Bucal.

[15] Castillo-Dalí G, Castillo-Oyagüe R, Terriza A, Saffar JL, Batista A, Barranco A (2014). In vivo comparative model of oxygen plasma and nanocomposite particles on PLGA membranes for guided bone regeneration processes to be applied in pre-prosthetic surgery: a pilot study. J Dent.

[16] Castillo-Dalí G, Castillo-Oyagüe R, Terriza A, Saffar JL, Batista-Cruzado A, Lynch CD (2016). Pre-prosthetic use of poly(lactic-co-glycolic acid) membranes treated with oxygen plasma and TiO(2) nanocomposite particles for guided bone regeneration processes. J Dent.

[17] Vowinckel J, Hartl J, Butler R, Ralser M (2015). MitoLoc: A method for the simultaneous quantification of mitochondrial network morphology and membrane potential in single cells. Mitochondrion.

[18] Liang M, Russell G, Hulley PA (2008). Bim, Bak, and Bax regulate osteoblast survival. J Bone Miner Res.

[19] Kapuściński J, Skoczylas B (1978). Fluorescent complexes of DNA with DAPI 4',6-diamidine-2-phenyl indole.2HCl or DCI 4',6-dicarboxyamide-2 phenyl indole. Nucleic Acids Res.

[20] Stuart KR, Cole ES (2000). Nuclear and cytoskeletal fluorescence microscopy techniques. Methods Cell Biol.

[21] Hotz MA, Traganos F, Darzynkiewicz Z (1992). Changes in nuclear chromatin related to apoptosis or necrosis induced by the DNA topoisomerase II inhibitor fostriecin in MOLT-4 and HL-60 cells are revealed by altered DNA sensitivity to denaturation. Exp Cell Res.

[22] Sharma P, Thummuri D, Reddy TS, Senwar KR, Naidu VG, Srinivasulu G (2016). New(E)-1-alkyl-1H-benzo[d]imidazol-2-yl)methylene)indolin-2-ones:Synthesis, in vitro cytotoxicity evaluation and apoptosis inducing studies. Eur J Med Chem.

[23] Dhurga DB, Suresh K, Tan TC (2016). Granular Formation during Apoptosis in Blastocystis sp. Exposed to Metronidazole (MTZ). PLoS One.

[24] Zhang Z, Li HM, Zhou C, Li Q, Ma L, Zhang Z (2016). Non-benzoquinone geldanamycin analogs trigger various forms of death in human breast cancer cells. J Exp Clin Cancer Res.

[25] Zreiqat H, Valenzuela SM, Nissan BB, Roest R, Knabe C, Radlanski RJ (2005). The effect of surface chemistry modification of titanium alloy on signaling pathways in human osteoblasts. Biomaterials.

[26] Salido M, Vilches JI, Gutiérrez JL, Vilches J (2007). Actin cytoskeletal organization in human osteoblasts grown on different dental titanium implant surfaces. Histol Histopathol.

[27] Yano K, Hoshino M, Ohta Y, Manaka T, Naka Y, Imai Y (2009). Osteoinductive capacity and heat stability of recombinant human bone morphogenetic protein-2 produced by Escherichia coli and dimerized by biochemical processing. J Bone Miner Metab.

[28] Bessa PC, Pedro AJ, Klösch B, Nobre A, van Griensven M, Reis RL (2008). Osteoinduction in human fat-derived stem cells by recombinant human bone morphogenetic protein-2 produced in Escherichia coli. Biotechnol Lett.

[29] Choi BD, Lee SY, Jeong SJ, Lim DS, Cha HJ, Chung WG (2016). Secretory leukocyte protease inhibitor promotes differentiation and mineralization of MC3T3-E1 preosteoblasts on a titanium surface. Mol Med Rep.

[30] Oliveira NC, Moura CC, Zanetta-Barbosa D, Mendonça DB, Cooper L, Mendonça G (2013). Effects of titanium surface anodization with CaP incorporation on human osteoblastic response. Mater Sci Eng C Mater Biol Appl.

[31] Tiainen H, Monjo M, Knychala J, Nilsen O, Lyngstadaas SP, Ellingsen JE (2011). The effect of fluoride surface modification of ceramic TiO2 on the surface properties and biological response of osteoblastic cells in vitro. Biomed Mater.

[32] Sahrmann P, Mohn D, Zehnder M, Stark WJ, Imfeld T, Weber FE (2014). Effect of direct current on surface structure and cytocompatibility of titanium dental implants. Int J Oral Maxillofac Implants.

[33] Han SH, Kim KH, Han JS, Koo KT, Kim TI, Seol YJ (2011). Response of osteoblast-like cells cultured on zirconia to bone morphogenetic protein-2. J Periodontal Implant Sci.

[34] Thoma DS, Benic GI, Muñoz F, Kohal R, Sanz Martin I, Cantalapiedra AG (2015). Histological analysis of loaded zirconia and titanium dental implants: an experimental study in the dog mandible. J Clin Periodontol.

[35] Osman RB, Swain MV, Atieh M, Ma S, Duncan W (2014). Ceramic implants (Y-TZP): are they a viable alternative to titanium implants for the support of overdentures? A randomized clinical trial. Clin Oral Implants Res.

[36] Aydın C, Yılmaz H, Bankoğlu M (2013). A single-tooth, two-piece zirconia implant located in the anterior maxilla: a clinical report. J Prosthet Dent.

[37] Pierres A (2002). , Benoliel A.M. and Bongrand P. Cell fitting to adhesive surfaces: A prerequisite to firm attachment and subsequent events. Eur. Cell Mater.

[38] Zigmond S (1996). H. Signal transduction and actin filament organization. Curr. Opin. Cell Biol.

[39] Galli C, Piemontese M, Lumetti S, Ravanetti F, Macaluso GM, Passeri G (2012). Actin cytoskeleton controls activation of Wnt/β-catenin signaling in mesenchymal cells on implant surfaces with different topographies. Acta Biomater.

